# Exposure Time Impact on the Geomechanical Characteristics of Sandstone Formation during Horizontal Drilling

**DOI:** 10.3390/molecules25112480

**Published:** 2020-05-27

**Authors:** Hany Gamal, Salaheldin Elkatatny, Abdulazeez Abdulraheem, Abdulaziz Al Majed

**Affiliations:** College of Petroleum Engineering & Geosciences, King Fahd University of Petroleum & Minerals, Dhahran 31261, Saudi Arabia; g201706870@kfupm.edu.sa (H.G.); aazeez@kfupm.edu.sa (A.A.); aamajed@kfupm.edu.sa (A.A.M.)

**Keywords:** geomechanical properties, barite-water-based drilling fluid, exposure time, sandstone formation, horizontal drilling

## Abstract

The rock geomechanical properties are the key parameters for designing the drilling and fracturing operations and for programing the geomechanical earth models. During drilling, the horizontal-section drilling fluids interact with the reservoir rocks in different exposure time, and to date, there is no comprehensive work performed to study the effect of the exposure time on the changes in sandstone geomechanical properties. The objective of this paper is to address the exposure time effect on sandstone failure parameters such as unconfined compressive strength, tensile strength, acoustic properties, and dynamic elastic moduli while drilling horizontal sections using barite-weighted water-based drilling fluid. To simulate the reservoir conditions, Buff Berea sandstone core samples were exposed to the drilling fluid (using filter press) under 300 psi differential pressure and 200 °F temperature for different exposure times (up to 5 days). The rock characterization and geomechanical parameters were evaluated as a function of the exposure time. Scratch test was implemented to evaluate rock strength, while ultrasonic pulse velocity was used to obtain the sonic data to estimate dynamic elastic moduli. The rock characterization was accomplished by X-ray diffraction, nuclear magnetic resonance, and scanning electron microscope. The study findings showed that the rock compression and tensile strengths reduced as a function of exposure time (18% and 19% reduction for tensile strength and unconfined compression strength, respectively, after 5 days), while the formation damage displayed an increasing trend with time. The sonic results demonstrated an increase in the compressional and shear wave velocities with increasing exposure time. All the dynamic elastic moduli showed an increasing trend when extending the exposure time except Poisson’s ratio which presented a constant behavior after 1 day. Nuclear magnetic resonance results showed 41% porosity reduction during the five days of mud interaction. Scanning electron microscope images showed that the rock internal surface topography and internal integrity changed with exposure time, which supported the observed strength reduction and sonic variation. A new set of empirical correlations were developed to estimate the dynamic elastic moduli and failure parameters as a function of the exposure time and the porosity with high accuracy.

## 1. Introduction

The new practices in well drilling as highly inclined, multilateral, and long horizontal section caused wellbore instability issues as a result of rock geomechanics alteration. Economically, wellbore instability issues can increase the total drilling cost by 10–20% and is responsible for an annual economic loss of $1–6 billion in the oil industry worldwide [1,2]. During drilling operations, the drilled well penetrates many subsurface rock formations such that the drilling fluids interact with the rock minerals. The mineralogical compositions of the rock formations and the chemical activity of the drilling fluids play a critical role in the rock–fluid interaction, and downhole drilling conditions such as temperature, pressure, and exposure time control the degree of interactions during drilling operations. Most wellbore instability problems were reported during the long exposure time during drilling operations [3,4]. Formation damage is another effect of long exposure time. Drilling for only 15 min with overbalanced pressure can reduce the well productivity by 6–10% due to damage mainly caused by filtrate invasion and interaction with the formation [5]. Davarpanah et al. [6] developed a numerical model for formation damage based on experimental sensitivity analysis that involved contact time of the rock and drilling mud (from 0.5 to 2.5 h). The study results showed that increasing the contact time resulted in increasing the formation damage as the rock pore throats and cracks were filled with the drilling mud. The precipitation of drilling fluid solids is one of the critical factors for formation damage, and therefore, the new research for utilizing formate fluids for the drilling operations showed less formation damage with the formate fluids due to low solid amounts in its composition in addition to compatibility with reservoir rock [7,8]. The drilling fluid components affected the rock wettability and permeability significantly [9]. The results of drilling fluid–rock interactions can potentially affect both the petrophysical and geomechanical properties of the drilled formations [10]. It is therefore important to consider the exposure time effects on rock–drilling fluid interaction on the design of drilling and completion programs to mitigate wellbore instability issues as much as possible.

The rock geomechanical properties are considered key input parameters for geomechanical earth models, drilling and completion design, and stimulation operations [11,12,13]. Rock Young’s modulus and Poisson’s ratio are necessary for stress evaluation [14]. Many studies utilized the dynamic moduli to estimate the static moduli to be used as inputs for the modeling purposes as the case of reservoir simulation and earth modeling [14,15]. The rock unconfined compressive strength (UCS) is the most significant parameter among these properties for evaluating rock geomechanical behavior [16], and the strength is controlled by many rock parameters such as rock porosity, internal friction angle, grains particle size, and the cohesive forces [17]. Young’s modulus (*E*) represents the rock stiffness as it is the measure of rock sample resistance against the compressional uniaxial stress. Poisson’s ratio (*υ*) is defined as the measure of the rotation of the lateral expansion to the longitudinal contraction of the rock sample. Lamé’s parameters (λ and *G*) are elastic moduli, where *G* is also known as the rigidity or shear modulus which describes the rock resistance against shear deformation. Bulk modulus (*K*) is one of the most important elastic moduli, *K* is defined as the ratio of hydrostatic stress to the volumetric strain, and the inverse of K is the rock compressibility. The uniaxial compaction modulus or oedometer modulus (*H*) represents the plane wave modulus or the compressional P-wave modulus. The elastic moduli *E, λ, G, K,* and *H* are all measured in the same units as stress units [18].

### Drilling Fluid–Rock Interactions

Several works studied the effects of drilling fluids interactions with shale formations [10,19]. The effect of drilling fluids on the geomechanical properties of sandstone is not well studied; a very limited work is found in the literature in this area [20]. Previous experimental studies on the interaction between shale rocks and water indicated a weakening effect on rock mechanical properties [21,22,23,24].

Each formation has distinguished values for its geomechanical parameters based on its lithology, rock properties, and fluid flow conditions such as pressure and temperature [25,26]. The rock deformation and changes in internal pore systems affect the propagation of the sonic compressional and shear waves (*Vp* and *Vs*) through the rock samples and, as a result, affect the elastic moduli [27]. The sonic wave velocity depends on the rock pore geometry and intrinsic rock properties. As the rock porosity increases, *Vp* and *Vs* decrease. Also, the velocities increase with increasing the effective pressure as the pressure will cause the rock compressibility and create good contact for the rock matrix. The rock saturation was found to affect the sonic wave velocity as the rock saturated with oil was found to increase the *Vp* but did not affect the *Vs* [28].

Xu et al. [15] showed that there is a strong relationship between rock UCS and *E*, where the relation degree changed from rock type to another as sandstone and mudstone and that is because of the rock lithology. Yadav et al. [29] performed an experimental work to address the change in geomechanical properties (Young’s modulus, Poisson’s ratio, and peak strength) of Berea sandstone and shale samples after interaction with water-based mud (WBM) and oil-based mud (OBM). Using the triaxial test, they found that OBM is better than WBM in preserving the shale strength. Kitamura and Hirose [30] studied the effect of distilled water on the strength of different sandstone types as Rajasthan, Shirahama, and Berea sandstone. They carried out the indentation test to evaluate the rock hardness. Ultrasonic wave velocities and UCS were performed, and the results showed that UCS and Young’s modulus increased when the porosity decreased.

Muqtadir et al. [31] studied the effect of fluid saturation on Scioto sandstone strength properties. Results showed that the rock samples that were saturated with brine (3 wt.% KCl) were significantly weaker than the oil-saturated samples. The UCS and the tensile strength (TS) of the brine-saturated samples decreased by 9% and 40%, respectively, while in oil-saturated samples, the reduction was 10% and 25%, respectively. Xu et al. [20] studied the effect of drilling fluid on tight sandstone hardness. WBM and OBM were used, and the results showed that, after two hours, sandstone hardness decreased rapidly by 22.9% with WBM and by 10.1% with OBM. However, after 2 h and up to 15 days, the hardness decreased to 33.1% with WBM while the hardness remained constant with OBM. For OBM, temperature change has only a little effect on the hardness while hardness decreased at a temperature above 122 °F (50 °C) for WBM.

Motra and Stutz [32] showed that the dynamic elastic moduli (*E, K*, and *G*) of the metamorphic rocks (quartz mica schist, and amphibolite) were found to be a function of pressure and temperature. The sonic data results showed that P and S wave velocities increased with pressure increase and decreased with temperature increase. Karakul [33] studied the change in the strength of the clay-bearing rock due to the effects of drilling fluids. The study used claystone and mudstone rock types to study the drilling fluid effect on rock strength. The results indicated that the polymer-based drilling fluid is recommended and that bentonite- or KCl-based mud as polymer-based mud did not affect the rock UCS and tensile strength, and hence, it will not enhance the instability issues.

Mohamed et al. [34] evaluated the effect of water-based mud (WBM) and oil-based mud (OBM) on the geomechanical properties of different core samples. The experiments were run for different exposure times between the mud and the rock samples (30 min, 1 day, and 2 days) under 300 psi differential pressure and 250 °F. The results showed that *UCS* decreased as the exposure time increased for limestone samples. Lamik et al. [35] presented a new rock strength parameter that can be derived from the drilling parameters while drilling or from the sonic slowness log. The parameter is very sensitive to the lithology and helped to identify the rock type and formations’ boundary. Bageri et al. [36] studied the effect of the drilled rock geomechanical properties on the drilling fluid properties as the cuttings from the drilled formations were mixed with the drilling fluids with different concentrations. The study showed that the cutting weight percentage in the total fluid, UCS, and E of the drilled sandstone affects the properties of the rheological properties of the drilling fluid.

The objective of this paper is to assess the change in the acoustic properties, dynamic elastic moduli of Buff Berea sandstone, and its failure parameters (UCS and TS) due to interaction with barite-weighted WBM for different exposure time. The new contributions of this study involve, for the first time, assessing the effect of exposure time on the changes in geomechanical properties of the Buff Berea sandstone, modifying the aging cell for the filtration loss apparatus to accommodate rock sample, using NMR and SEM analysis to detect the effect of the exposure time after the interaction process with the drilling fluid, and integrating the petrophysical-geomechanical with statistical analysis to develop new sets of correlations that can be used to predict the geomechanical properties as a function of exposure time and porosity reduction.

## 2. Materials and Methods

For this study, Buff Berea sandstone samples were used and the drilling fluid was barite-weighted WBM with 12.25 pounds-per-gallon (ppg) density. The rock samples were characterized by performing routine core analysis (RCA), nuclear magnetic resonance (NMR), scanning electron microscope (SEM), and X-ray diffraction (XRD). RCA was conducted to estimate the bulk density, porosity, and permeability of the sandstone samples. The porosity and permeability were determined using the helium gas expansion porosimeter and NMR rock analyzer. NMR experiments were conducted to measure the porosity and pore size distribution of the cores. XRD was used to determine the samples’ mineralogical composition, which is a critical factor that controls rock mechanical properties. Scratch tests were conducted to obtain the *UCS*. Ultrasonic pulse velocity (UPV) was used to obtain the sonic velocities, which can be used to calculate the dynamic elastic moduli of the core samples (*E, υ, λ, G, K*, and *H*) as the change in wave velocities controls the rock geomechanical properties [32]. The experimental work procedures were performed as follows:

- Core samples were cut, and end face grinding was accomplished.
- Sister core samples were selected as a reference.
- Samples were saturated with 3 wt.% KCl for clay stabilization.
- Filtration tests were performed under 200 °F and differential pressure of 300 psi to simulate the reservoir conditions.
- The acoustic data was determined.
- Rock characterization (NMR and SEM).
- Scratch test was performed to get the UCS.

The filtration tests in step 4 were performed for different runs (30 min and 1, 3, and 5 days) to address the effect of the exposure time. NMR, SEM, and UPV were run for the saturated samples before and after the interaction with WBM using the high-pressure high-temperature (HPHT) filtration cell. Scratch test was performed on the saturated sample (sister core samples) as it is partially destructive for the core sample, and after, filtration test was performed for each sample.

### 2.1. Core Samples Preparation and Characterization

The core samples were cut into 1.5′′ diameter and 2′′ length cylinders for use in the modified aging cell of the filtration test. The end surfaces were ground to obtain a very uniform sample length and diameter. The samples were then saturated by vacuum saturation method with 3 wt.% KCl to prevent clay swelling.

The Buff Berea mineralogical composition was obtained using X-ray diffraction (XRD). Buff Berea core samples have a bulk density of 2.07 g/cm^3^. The average porosity was 20.28% with a standard deviation of 0.26, while the permeability was 150.77 mD with a 2.05 standard deviation. Table 1 lists the XRD results of the rock sample as a component compositional percentage. XRD analysis indicated that the quartz content represented 94 wt.% and that microcline (alkali feldspar) content was 4.25 wt.% while albite (plagioclase feldspar) with 1 wt.% and the sample had a very low content of calcite, rutile, and biotite. Microcline and albite represented the clay minerals which are commonly composed of aluminum silicates that are linked to each other through the sharing of apical oxygen atoms [37]. Any chemical interactions that lead to the dissolution of sandstone can cause a change in the rock properties [38]. The clay minerals can decompose when it is exposed to water [39].

### 2.2. Drilling Fluid Preparation and Rheology Measurements

Barite-weighted WBM with 12.25 ppg density was prepared using the compositions shown in Table 2. Water (290 g) was used as the base fluid for the mud formulation, while xanthan gum (XC) polymer and bentonite were used as viscosifiers. Starch was used as the fluid loss control agent, while potassium chloride (KCl) served as a clay anti-swelling agent. Potassium hydroxide (KOH) serves as the pH controller, calcium carbonate (CaCO_3_) with a medium size (D_50_ of 50 microns) serves as a bridging agent, and barite was used as a weighting material to provide the desired mud density.

After preparing the drilling fluid, the fluid density and rheological properties were measured at atmospheric pressure and room temperature (80 °F). Mud balance was used to obtain the mud density, and 900-Viscometer^®^ was used to determine the shear stress at different shear rates. Table 3 shows that the prepared mud had a density of 12.35 ppg; 13 cP plastic viscosity; 63 lb/100 ft^2^ yield point; and gel strengths of 11, 21, and 21 lb/100 ft^2^ for 10-s, 10-min, and 30-min readings, respectively.

### 2.3. Rock-Fluid Interaction (Filtration Test)

**Figure 1:**
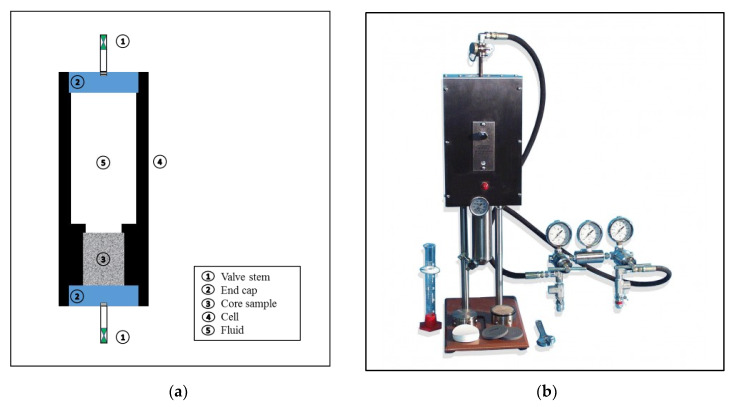
Filtration test equipment: (**a**) Modified filter press cell diagram; (**b**) high-pressure high-temperature (HPHT) filter press.

Filtration experiments were conducted using a filter press cell that was modified to be able to accommodate a core sample with 1.5” diameter and 2” length as shown in Figure 1a. The core sample was placed in the cell, and the drilling fluid was poured in the filtration cell. The cell was placed in the filter press jacket (Figure 1b). To simulate the reservoir conditions, the cell was heated up to 200 °F under a differential pressure of 300 psi. The filtrate volume was recorded as a function of time for up to 30 min. The test was repeated for different filtration times, lasting up to 5 days to study the effect of extended time on rock–fluid interaction. The objective was to mimic the condition of drilling long horizontal sections whereby the drilling mud has an extended contact and interaction with the reservoir rock for as long as 20 days of drilling.

### 2.4. Acoustic Velocities and Dynamic Elastic Moduli

**Figure 2:**
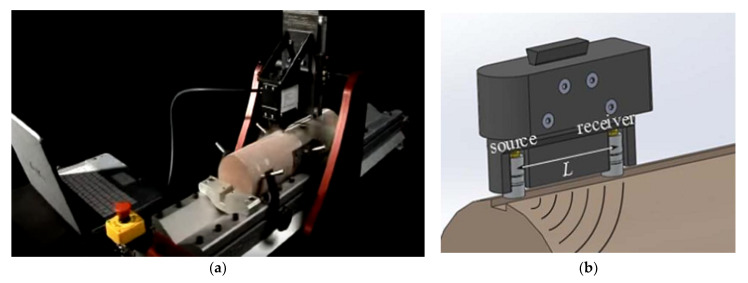
Scratch-test machine used in the study: (**a**) unconfined compressive strength (UCS) evaluation; (**b**) ultrasonic velocity acquisition.

The scratch machine (Figure 2a) was used to acquire strength and sonic data. Two probes (one transmitter and one receiver with a spacing of 2 inches) are fixed in the place of the cutting tool in the scratch test machine (Figure 2b). The combination of the continuous profiles of rock strength UCS and ultrasonic velocity contributes to the identification of different geomechanical parameters [40].

The Ultrasonic pulse velocity test (UPV) was conducted to acquire the compressional and shear velocities (*V_P_* and *V_S_*). Dynamic Young’s modulus and Poisson’s ratio were determined by the prorogation of pressure P and shear S waves in the core samples. According to the American society for testing and materials standard method [41], dynamic Young’s modulus (*E_d_*) and dynamic Poisson’s ratio (*v_d_*) can be calculated from *V_P_*, *V_S_*, and the density (ρ) of the core samples by Equations (1) and (2), respectively.

(1) $$E_{d}=\frac{\rho V_{S}^{2}\left(3V_{P}^{2}-4V_{S}^{2}\right)}{V_{P}^{2}-V_{S}^{2}}$$

(2) $$\upsilon_{d}=\frac{V_{P}^{2}-2V_{S}^{2}}{2*\left(V_{P}^{2}-V_{s}^{2}\right)}$$

The other dynamic moduli were calculated using the following equations [18]:

(3) $$K=\frac{E}{3\;\left(1-v\right)}$$

(4) $$G=\frac{E}{2\;\left(1+v\right)}$$

(5) λ=H−2G

(6) $$H=\frac{3K\left(1-v\right)}{\;\left(1+v\right)}$$

### 2.5. Scratch Testing for Rock Strength

The unconfined compressive strength (UCS) was measured using a scratch testing machine (Figure 2a). Scratch testing is considered a practical technique to determine rock strength. The mechanism for the test involves using a sharp cutter tool to scratch the rock surface with a depth, typically 1 mm, while monitoring the applied forces (shear and normal forces). The applied forces are proportional to the rock-specific energy that correlates to the core strength UCS such that the test provides a continuous strength profile along the core length. This method has been applied to different research works [42,43]. The scratch test method is quick, partially destructive, and inexpensive. It does not need extensive core preparation, and a continuous strength data profile along the core length can be acquired [44].

The rock tensile strength (TS) represents the rock ability to resist the failure, and it is an important rock property for the rock fracturing jobs [45]. There are two standard laboratory methods to determine the rock tensile strength which are the direct and indirect Brazilian methods [46,47,48]. Many studies were performed to correlate the rock UCS and TS for different rock types [45,49,50]. Altindag and Guney [49] used data for 143 samples of different rock types and get the following correlation (with correlation coefficient (R) of 0.9)

TS = 0.0963 UCS^0.932^ (7)

where UCS and TS are in mega Pascal (MPa).

### 2.6. Scanning Electron Microscopy (SEM) and Nuclear Magnetic Resonance (NMR)

SEM with EDS (energy-dispersive X-ray spectroscopy) was performed on sections from the rock samples before and after the mud interaction to study the rock integrity in terms of cementing, the internal surface topography, and the composition of the samples. Mud-induced formation damage was studied using several laboratory techniques such as X-ray diffractions and scanning electron microscopes [51]. SEM has been used in many types of research works to determine fines deposition and internal system changes at micro- and nanometer scales [52,53,54].

NMR was used to characterize the internal pore structure through measurements of the T_2_ relaxation of the protons in the water saturating the rock pores. T_2_ is a time constant that describes the relaxation rate of the protons after they were polarized by an external magnetic field. Nuclear magnetic resonance (NMR) relaxometry technique was used to identify mud-induced damage to rock pore systems [55,56,57,58]. In this study, NMR measurements were conducted before exposure and after different exposure times to determine the corresponding porosity and pore size distribution resulting from the rock–fluid interaction.

## 3. Results and Discussion

The rock–mud interaction was executed through the filtration test under the designed pressure and temperature and with different exposure times. The filtrate volume during the filtration test was recorded for 30 min following the API standard [59]. The results showed that an average of 5.5 cm^3^ was collected during the 30 min under 200 °F and 300 psi differential pressure. The filtration test indicates the flow properties of the drilling fluid through the rock medium. During the test, the mud starts to formulate the mud cake; in the same time, the drilling fluid and its solids invade the rock sample pores by the action of pressure applied, and as a result, the rock pore system will change and the filtrate fluid will interact with the rock mineral composition.

### 3.1. Effect of Exposure Time on the Acoustic Waves

The sonic measurements with the exposure time were recorded and showed an increase in the *Vp* and *Vs* with increasing the time of mud interaction. The reason behind the increase in the sonic wave velocities with extending exposure time was the change in the internal pore system as the rock porosity decreased by mud solids invasion as confirmed by NMR results. The change in the internal rock pore system affected the wave propagation velocity. The *Vp* recorded 2304 m/s after the 30 min and increased with time to record 2425 m/s after the 5 days mud interaction. After the first exposure time (30 min), *Vs* was 1250 m/s and increased to 1305 m/s after 5 days. Table 4 summarizes the recorded sonic data.

**Figure 3:**
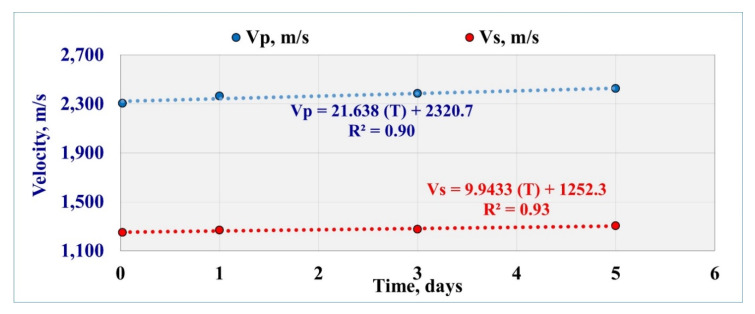
Sonic measurements after the filtration test with different exposure time.

Figure 3 represents that *Vp* and *Vs* increased with a linear relationship with time, where R^2^ showed 0.9 for *Vp* and 0.93 for *Vs*:

*Vp* = 21.638 (*T*) + 2320.7 (8)

*Vs* = 9.9433 (*T*) + 1252.3 (9)

### 3.2. Effect of Exposure Time on the Elastic Parameters (E_d_ and v_d_)

**Figure 4:**
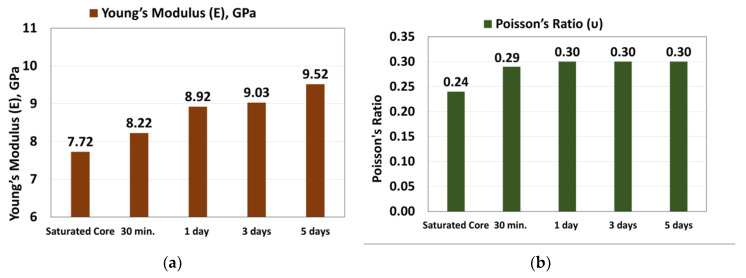
Exposure time effect on (**a**) Young’s modulus and (**b**) Poisson’s ratio.

Figure 4 showed that Young’s modulus increased from 7.72 GPa for the saturated rock and increased to record 9.52 after five days of mud interaction (23% increase percentage) (Figure 4a). Poisson’s ratio was 0.24 for the saturated rock and increased with the mud interaction to 0.29 after 30 min, and it was stabilized at 0.3 after one day and then did not change (Figure 4b).

### 3.3. Effect of Exposure Time on Other Geomechanical Parameters

**Figure 5:**
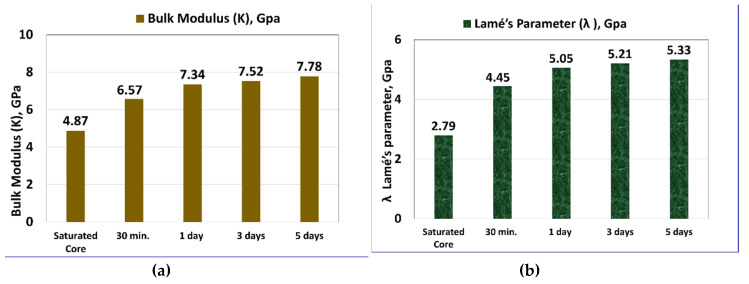

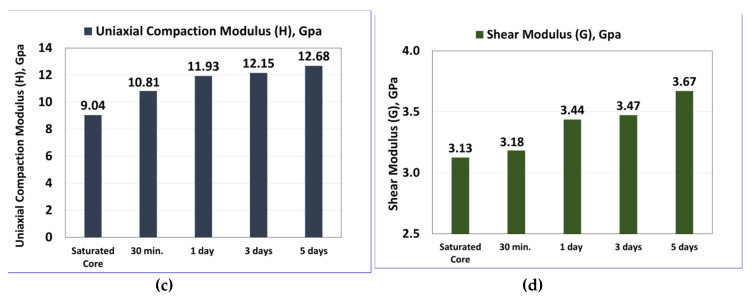
Geomechanical elastic moduli for the samples with extended exposure time: (**a**) *K*, (**b**) *λ*, (**c**) *H*, and (**d**) *G*.

As shown in Figure 5, the other dynamic elastic moduli (*K, λ, G*, and *H*) showed an increase with increasing exposure time. After five days of mud interaction, *K* increased from 4.87 to 7.78 GPa with 60% increase (Figure 5a), *λ* increased from 2.79 to 5.33 GPa (Figure 5b), *H* value increased by 40% as it increased from 9.04 to 12.68 GPa (Figure 5c), while *G* increased from 3.13 to 3.67 GPa (17% increase) (Figure 5d). Table 5 summarizes the calculated geomechanical properties with extended exposure time. Table 6 shows the dynamic moduli correlations as a function of the exposure time using the regression analysis.

### 3.4. Effect of Exposure Time on Failure Parameter (UCS and TS)

Scratch test was used to evaluate the rock strength for all the investigated exposure times. Table 7 represents the *UCS* and *TS* results for the Buff Berea sandstone samples at 100% saturated sample and then for 30 min, 1 day, 3 days, and 5 days of filtration and rock–fluid interaction. The UCS appears to decrease as the interaction time increases. The pre-infiltration value of UCS was 50.25 MPa and then decreased to 40.51 MPa after 5 days of interactions with a reduction percentage of 19%. The *UCS* decreases by 2% after 30 min of rock–fluid interaction and then by 9% after 1 day of interaction to record 45.9 MPa. After 3 days, UCS recorded 41.5 MPa (17% reduction percentage). The results showed that rock strength decreased drastically after the third day and remained unchanged afterward.

The TS was estimated from the UCS–TS correlation (Equation (7)), and the results showed that the TS was 3.71 MPa for the saturated state and decreased to record 3.64 MPa after 30 min of mud interaction and that TS reduced to 3.41 MPa after 1 day, to 3.11 MPa after three days, and finally to 3.04 MPa after the 5th day.

**Figure 6:**
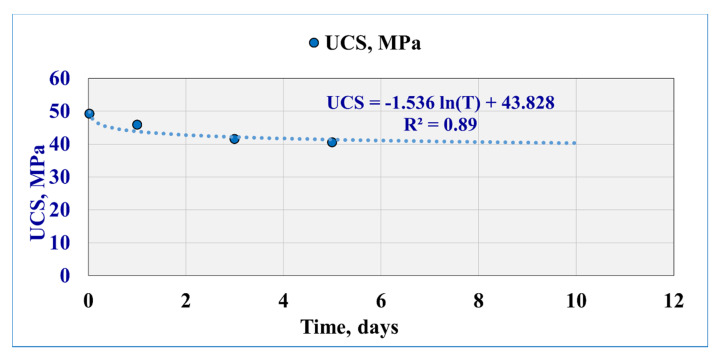
Strength alteration with an extended time of interaction with barite-weighted WBM.

From the rock UCS and TS measurements, the results showed rock strength reduction with time as a weakening effect; statistical analysis for the results of extending the exposure time (from 30 min to 5 days) was performed and showed that there is a logarithmic relationship with 0.89 coefficient of determination between the UCS values with the extended exposure time (Figure 6). Extending the exposure time up to 10 days based on the correlation shows that the UCS value is 40.3 MPa, which means that the UCS reduction might stabilize under the current operating conditions. UCS and TS are time-dependent as the following correlations:

UCS = −1.536 ln(T) + 43.828(10)

TS = −0.107 ln(T) + 3.2674(11)

### 3.5. Alteration Mechanism (SEM and NMR Results)

**Figure 7:**
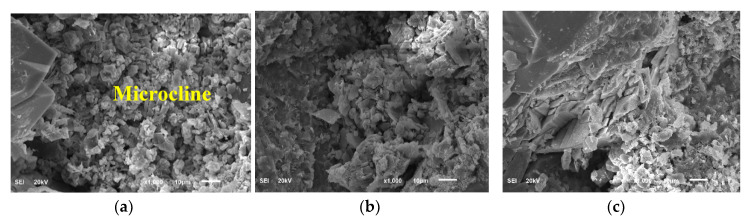

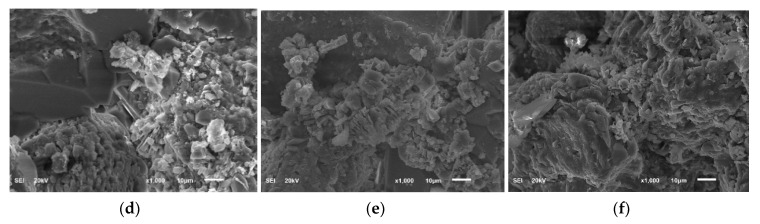
SEM results: (**a**) Dry sample; (**b**) saturated sample; (**c**) after 30 min of interaction; (**d**) after 1 day of interaction; (**e**) after 3 days of interaction; and (**f**) after 5 days of interaction.

SEM analysis was conducted to acquire information on the rock surface topography and composition at different states of rock–fluid interaction. Figure 7 shows the SEM images of the Buff Berea sample at different states from the dry condition through saturation (3 wt.% KCl) and then the different times of interaction with barite-weighted WBM. The results show the changes in the internal surface topography of the rock samples during each condition. As shown, microcline (clay mineral) appears to swell and the swelling increases with increasing exposure time with the drilling fluid. The swelling and possible destruction and redistribution of clay minerals may be responsible for the change in the geomechanical properties of the rock as explained in geomechanics and strength sections. Ombaka [60] also highlighted that disturbance in clay minerals can cause changes in the rock cohesion, swelling, and plasticity. 

**Figure 8:**
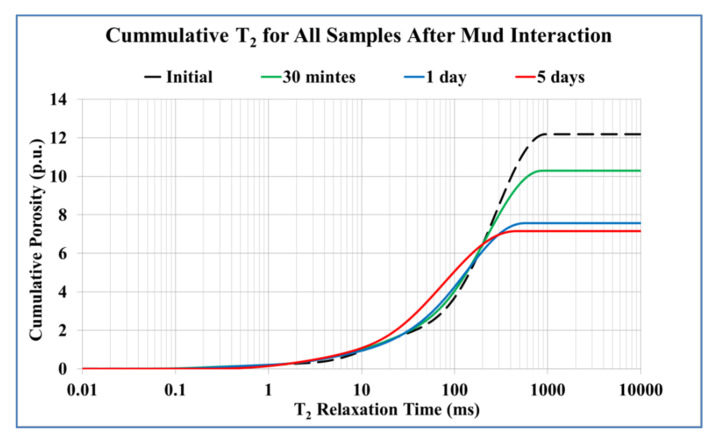
Cumulative T_2_ for the core samples after the interaction with the drilling fluid.

Figure 8 shows the probability distribution function (PDF) plot of the *T_2_* data, which is a representation of the pore size distribution in a rock system: PDF of *T_2_* relaxation time for the core samples as saturated and after the different exposure times of mud interaction (after 30 min of filtration, after 1 day, after 3 days, and after 5 days). The chart shows that the porosity of the rock decreased as the filtration time increases due to the invasion of mud solids into the rock pore system.

The NMR results showed that the total porosity decreased from the initial value of 21% down to 12.4% after the 5th day of mud interaction with 41% reduction percentage from the initial porosity. After 30 min of mud interaction, the porosity recorded 17.6% and reached 13.2% after 1 day (37% porosity reduction). The porosity showed 12.8% after the 3rd day with a 39% reduction percentage. Hence, it can be observed that the porosity reduced drastically after 30 min and 1 day, while the reductions in the subsequent days were minimal.

**Figure 9:**
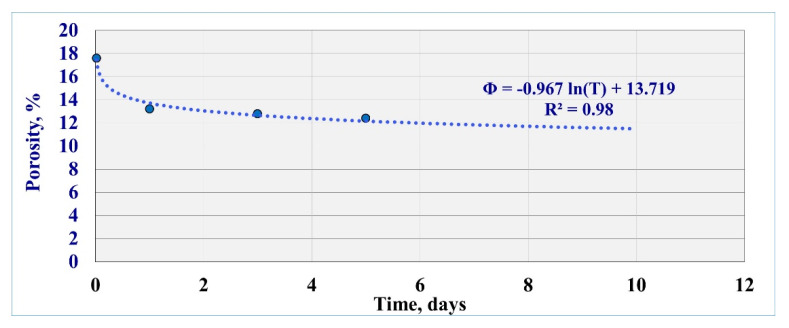
Porosity reduction as a function of the extended exposure time.

Figure 9 represents the formation of damage that occurred as a function of exposure time. The figure shows the porosity reduction as time-dependent.

The rock porosity showed a logarithmic regression relationship with a coefficient of determination (R^2^) of 0.98 between the porosity (*Φ*) and the extended exposure time (*T*) as follows:

*Φ =* −0.967*ln(T)* + 13.719 (12)

Extending the exposure time to 10 days, the porosity will record 11.5%, which represents the approximate stabilization.

### 3.6. Development of New Correlations for Geomechanical Parameters

The regression analysis is considered a statistical technique that is usually used to present the relation between the parameter of interest and the variable parameters. The obtained results were used to develop the correlations between the petrophysical-geomechanical parameters, the porosity, and the exposure time using multiple regression techniques. The regression analysis showed that the rock strength and dynamic elastic moduli can be estimated by the obtained correlations from the nonlinear regression analysis (with R^2^ from 0.96 to 1.0) as shown in Table 8.

The outputs from the study explained the exposure time effect on the rock elastic and failure properties for sandstone. SEM represented how the interaction affected the internal surface topography by increasing the time of rock–mud interaction. The microcline as clay materials displayed a swelling effect because of the invaded filtrate into the rock; the behavior increased with increasing the exposure time; and therefore, the rock cohesion and integrity changed and affected the rock strength as it might cause changes in the bonding forces between the solid particles [24]. The internal topography changes affect the propagation of the sonic waves (*Vp* and *Vs*) through the rock samples, and as a result, there is an alteration in the rock geomechanics in terms of the dynamic elastic moduli [32]. The NMR cumulative *T_2_* results showed a reduction in the porosity system with time because of barite particles precipitations and swelling effect.

## 4. Conclusions

This study presents extensive laboratory works aimed to assess the changes in the geomechanical properties of Buff Berea sandstone rock samples subjected to different interaction times with the water-based drilling fluid. Based on the obtained results, the following conclusions are made.

- The rock samples showed formation damage increase with increasing exposure time, as the porosity showed sever reduction after a one-day interaction with 37% porosity reduction, while 41% reduction was found after five days of interaction; the clay swelling and mud solid invasion were the reasons behind that damage.
- The strength reduction was observed as the UCS decreased from 50.25 MPa before the mud interaction and then decreased to 40.51 MPa after five days of mud interactions with (19% UCS reduction). TS decreased from 3.71 to 3.04 MPa (18% reduction) within the five days of mud exposure.
- The rock dynamic elastic moduli showed an increasing trend as E increased from 7.72 GPa before mud interaction to 9.52 GPa 9.52 after five days exposure time (23% increase percentage). K increased from 4.87 to 7.78 GPa with a 60% increase, G increased from 3.13 to 3.67 GPa (17% increase), and H value increased from 9.04 to 12.68 GPa (40% increase).
- A new set of empirical correlations was developed to estimate the dynamic elastic moduli and failure parameters as a function of the exposure time and the porosity with high accuracy.

## Figures and Tables

**Table 1:** X-ray diffraction (XRD) of Buff Berea sandstone rock sample.

Mineral	Chemical Symbol	Concentration (wt.%)
Quartz	SiO_2_	94
Microcline	KAlSi_3_O_8_	4.25
Albite	NaAlSi_3_O_8_	1
Calcite	CaCO_3_	0.4
Rutile	TiO_2_	0.25
Biotite	K(Mg,Fe++)_3_[AlSi_3_O_10_(OH,F)]_2_	0.1

**Table 2:** Barite-weighted water-based mud (WBM) formulation.

Material	Amount (g)	Function
Water	290	Base
Defoamer	0.08	Anti-foam agent
XC-polymer	1.5	Viscosifier
Bentonite	4	Viscosifier
Starch	6	Fluid loss control
KCl	20	Clay stabilization
KOH	0.3	pH control
CaCO_3_	5	Bridging agent
Barite	200	Weighting material

**Table 3:** Mud Properties at 80 °F.

Mud Property at 80 °F		Unit
Density	12.35	ppg
Plastic Viscosity	13	cP
Yield Point	63	lb/100 ft^2^
Gel strength after 10 s	11	lb/100 ft^2^
Gel strength after 10 min	21	lb/100 ft^2^
Gel strength after 30 min	21	lb/100 ft^2^

**Table 4:** The sonic data recorded at each condition.

Sample Condition	*Vp* (m/s)	*Vs* (m/s)
100% Saturated	2109	1240
30 min of Interaction	2304	1250
1 day of Interaction	2364	1269
3 days of Interaction	2385	1275
5 days of Interaction	2425	1305

**Table 5:** Summary of the geomechanical elastic moduli with the exposure time.

Sample Condition	$E_{d},\;\mathrm{GPa}$	$\upsilon_{d}\;$	*K*, Gpa	*G*, Gpa	*H*, Gpa	*λ*, Gpa
100% Saturated	7.72	0.24	4.87	3.13	9.04	2.79
30-min Interaction	8.22	0.29	6.57	3.18	10.81	4.45
1-day Interaction	8.92	0.3	7.34	3.44	11.93	5.05
3-day Interaction	9.03	0.3	7.52	3.47	12.15	5.21
5-day Interaction	9.52	0.3	7.78	3.67	12.68	5.33

**Table 6:** Dynamic elastic moduli as a function of exposure time.

H = 0.31 ln(T) + 11.983
E = 0.2052 ln(T) + 8.9813
K = 0.2086 ln(T) + 7.3651
λ = 0.1579 ln(T) + 5.0564
G = 0.0761 ln(T) + 3.4631

**Table 7:** Summary of the strength alteration with the extended time of mud interaction.

Sample Condition	UCS (MPa)	TS (MPa)
100% Saturated	50.25	3.71
30 min of Interaction	49.24	3.64
1 day of Interaction	45.85	3.41
3 days of Interaction	41.5	3.11
5 days of Interaction	40.51	3.04

**Table 8:** New correlations for geomechanical parameters as a function of exposure time and porosity.

Parameter	Correlation
UCS	$\mathrm{UCS}=39.365*\;0.972^{T}*\;1.013^{\Phi }$
TS	$\mathrm{TS}=2.957*\;0.974^{T}*\;1.012^{\Phi }$
$E_{d}$	$E_{d}=10.515*\;1.013^{T}*\;0.986^{\Phi }$
K	$K=9.800*\;1.010^{T}*\;0.978^{\Phi }$
G	$G=3.973*\;1.014^{T}*\;0.987^{\Phi }$
H	$H=15.055*\;1.012^{T}*\;0.981^{\Phi }$
λ	$\lambda =7.205*\;1.008^{T}*\;0.973^{\Phi }$

## Nomenclature

**Symbol**	**Parameter**	**Unit**
UCS	Unconfined compressive strength	MPa
TS	Tensile strength	MPa
WBM	Water-based mud	
OBM	Oil-based mud	
UPV	Ultrasonic pulse velocity	
XRD	X-ray diffraction	
NMR	Nuclear magnetic resonance	
SEM	Scanning electron microscope	
RCA	Routine core analysis	
EDS	Energy-dispersive spectroscopy	
HPHT	high-pressure high-temperature	
R^2^	Coefficient of determination	
R	Correlation coefficient	
MEM	Mechanical earth modeling	
T	Exposure time	Day
Φ	Porosity	%
Vp	Compressional wave velocity	m/s
Vs	Shear wave velocity	m/s
υ	Poisson’s ratio	GPa
λ	Lamé’s parameters	GPa
G	Rigidity or shear modulus	GPa
K	Bulk modulus	GPa
H	Uniaxial compaction or oedometer modulus	GPa
PDF	Probability distribution function	
T_2_	Relaxation time	

## References

[1] Kang Y., Yu M., Miska S.Z., Takach N. Wellbore Stability: A Critical Review and Introduction to DEM. Proceedings of the Annual Technical Conference and Exhibition.

[2] Albukhari T.M., Beshish G.K., Abouzbeda M.M., Madi A. Geomechanical Wellbore Stability Analysis for the Reservoir Section in JNC186 Oil Field. Proceedings of the ISRM 1st International Conference on Advances in Rock Mechanics—TuniRock.

[3] Al-Buraik K.A., Pasnak J.M. Horizontal Drilling in Saudi Arabian Oil Fields: Case Histories. Proceedings of the Middle East Oil Show.

[4] Bakar M.A.A., Nasrudin K.A., Nor M.A.M., Mclellan J., Bradbury A., Syrapushchynski S., Rockwood J. Application of a Wellbore Shield Eliminates NPT in South China Sea Wells. Proceedings of the SPE/IADC Middle East Drilling Technology Conference and Exhibition.

[5] Ding Y., Herzhaft B., Renard G. Near-Wellbore Formation Damage Effects on Well Performance—A Comparison between Underbalanced and Overbalanced Drilling. Proceedings of the SPE International Symposium and Exhibition on Formation Damage Control.

[6] Davarpanah A., Mirshekari B., Razmjoo A. (2020). A parametric study to numerically analyze the formation damage effect. Energ. Explor. Exploit..

[7] Davarpanah A. (2019). The feasible visual laboratory investigation of formate fluids on the rheological properties of a shale formation. Int. J. Environ. Sci. Technol..

[8] Davarpanah A., Mirshekari B. (2019). Effect of formate fluids on the shale stabilization of shale layers. Energy Rep..

[9] Sharma M.M., Wunderlich R.W. (1987). The Alteration of Rock Properties Due to Interactions with Drilling-Fluid Components. J. Petroleum Sci. Eng..

[10] Chukwuemeka A.O., Amede G., Alfazazi U. (2017). A Review of Wellbore Instability during Well Construction: Types, Causes, Prevention and Control. Pet. Coal.

[11] Yale D.P., Jamieson W.H. Static and dynamic mechanical properties of carbonates. Proceedings of the American Rock Mechanics Association Source 1st North American Rock Mechanics Symposium.

[12] Larsen I., Fjrer E., Renlie L. Static and dynamic Poisson’s ratio of weak sandstones. Proceedings of the 4th North American Rock Mechanics Symposium.

[13] Elkatatny S.M., Tariq Z., Mahmoud M.A., Abdulraheem A., Abdelwahab A.Z. An Artificial Intelligent Approach to Predict Static Poisson’s Ratio. Proceedings of the 51st US Rock Mechanics/Geomechanics Symposium.

[14] Zheng M., Tang H., Li H., Zheng J., Jing C. (2020). Geomechanical Analysis for Deep Shale Gas Exploration Wells in the NDNR Blocks, Sichuan Basin, Southwest China. Energies.

[15] Xu H., Zhou W., Xie R., Da L., Xiao C., Shan Y., Zhang H. (2016). Characterization of rock mechanical properties using lab tests and numerical interpretation model of well logs. Math. Probl. Eng..

[16] Romana M., Vásárhelyi B. A Discussion on the Decrease of Unconfined Compressive Strength between Saturated and Dry Rock Samples. Proceedings of the International Society for Rock Mechanics and Rock Engineering 11th ISRM Congress.

[17] Zoback M.D. (2010). Reservoir Geomechanics.

[18] Fjaer E., Holt R.M., Raaen A.M., Risnes R., Horsrud P. (2008). Petroleum Related Rock Mechanics. Borehole Failure Criteria.

[19] Steiger R.P., Leung P.K. (1992). Quantitative Determination of the Mechanical Properties of Shales. SPE Drill. Eng..

[20] Xu F., Yan Z., Wang L., Guo Y., Yang C. (2018). Effect of the Drilling Fluid on Hardness Characteristics of Tight Sandstone. Curr. Sci..

[21] Han G. (2013). Rock Stability under Different Fluid Flow Conditions. Ph.D. Thesis.

[22] Meng Z.P., Wu Y., Tiedemann J. Analysis of Mechanical Properties of Sedimentary Rocks of Coal Measures and Their Influencing Factors. Proceedings of the 40th U.S. Symposium on Rock Mechanics (USRMS).

[23] Lin S., Lai B. Experimental Investigation of Water Saturation Effects on Barnett Shale Geomechanical Behaviors. Proceedings of the SPE Annual Technical Conference and Exhibitionr.

[24] Hu R., Liu H.H., Chen Y.F., Zhou C.B., Gallipoli D. (2014). A Constitutive Model for Unsaturated Soils with Consideration of Inter-particle Bonding. Comput. Geotech..

[25] Ameen M.S., Smart B.G.D., Somerville J.M., Hammilton S., Naji N.A. (2009). Predicting rock mechanical properties of carbonates from wireline logs (A case study: Arab-D reservoir, Ghawar field, Saudi Arabia). Mar. Pet. Geol..

[26] Al-Anazi A., Gates I.D. (2010). A Support Vector Machine Algorithm to Classify Lithofacies and Model Permeability in Heterogeneous Reservoirs. Eng. Geol..

[27] Wang Z., Hirsche W.K., Sedgwick G. (1991). Seismic velocities in carbonate rocks. J. Can. Petrol. Technol..

[28] Lee M.W. (2003). Velocity Ratio and Its Application to Predicting Velocities.

[29] Yadav P.K., Ali S.S., Tawat N.A.A., Dhamen A.A.A., Jin G. Effect of Drilling Fluid on Rock Mechanical Properties at Near-Drilling Conditions: An Implication of Fluid Design on Wellbore Stability. Proceedings of the Technology Conference Asia.

[30] Kitamura M., Hirose T. (2017). Strength Determination of Rocks by Using Indentation Tests with a Spherical Indenter. J. Struct. Geol..

[31] Muqtadir A., Elkatatny S.M., Mahmoud M.A., Abdulraheem A., Gomaa A. Effect of Saturating Fluid on the Geomechanical Properties of Low Permeability Scioto Sandstone Rocks. Proceedings of the 52nd US Rock Mechanics/Geomechanics Symposium.

[32] Motra H.B., Stutz H.H. (2018). Geomechanical rock properties using pressure and temperature dependence of elastic P-and S-wave velocities. Geotech. Geol. Eng..

[33] Karakul H. (2018). Effects of drilling fluids on the strength properties of clay-bearing rocks. Arab. J. Geosci..

[34] Mohamed A.K., Benaafi M., Elkatatny S.M., Bageri B.S. Effect of High-Density Water-Based Drilling Fluid on the Mechanical Properties of the Drilled Formation in Horizontal Wells. Proceedings of the 53rd US Rock Mechanics/Geomechanics Symposium.

[35] Lamik-Thonhauser B., Schoen J.H., Koller C.S., Arnaout A.M. Correlation between Drilling Parameters and Geomechanical Properties—The Hidden Geomechanical Information. Proceedings of the Abu Dhabi International Petroleum Exhibition and Conference.

[36] Bageri B.S., Benaafi M., Mahmoud M., Mohamed A., Patil S., Elkatatny S. (2020). Effect of Formation Cutting’s Mechanical Properties on Drilling Fluid Properties During Drilling Operations. Arab. J. Sci. Eng..

[37] Madejová J. (2003). FTIR techniques in clay mineral studies. Vib. Spectrosc..

[38] Baiyegunhi C., Liu K., Gwavava O. (2017). Diagenesis and reservoir properties of the permian Ecca Group sandstones and mudrocks in the Eastern Cape Province, South Africa. Minerals.

[39] Armstrong L.C. (1940). Decomposition and alteration of feldspars and spodumene by water. American Mineralogist. J. Earth Planet. Mater..

[40] Noufal A., Germay C., Lhomme T., Thomas R. Uncertainty Reduction in Geomechanical Modeling Using Continuous Core Based Data in a Giant Field, Abu Dhabi, UAE. Presented at the Abu Dhabi International Petroleum Exhibition & Conference.

[41] ASTM D2845-00 (2000). Standard Test Method for Laboratory Determination of Pulse Velocities and Ultrasonic Elastic Constants of Rock.

[42] Schei G., Fjær E., Detournay E., Kenter C.J., Fuh G.F., Zausa F. The Scratch Test: An Attractive Technique for Determining Strength and Elastic Properties of Sedimentary Rocks. Presented at the SPE Annual Technical Conference and Exhibition.

[43] Garcia P.F.V., Rossi D.F., Ferreira F.H., Santos E.S.R., Borba A.M. Mechanical Characterization of Grainstone and Dolomite Rock Samples from Quissamã Formation, Campos Basin. Presented at the Offshore Technology Conference.

[44] Germay C., Richard T. The Scratch Test: A High Resolution Log of Rock Strength with Application to Geomechanic and Petrophysic. Presented at the Society of Petrophysicists and Well-Log Analysts SPWLA 55th Annual Logging Symposium.

[45] Nazir R., Momeni E., Armaghani D.J., Amin M.M. (2013). Correlation between unconfined compressive strength and indirect tensile strength of limestone rock samples. Electron. J. Geotech. Eng..

[46] ISRM (1978). Suggested methods for determining tensile strength of rock materials. Int. J. Rock Mech. Min. Sci. Geomech. Abstr..

[47] ASTM (2008). D2936-08: Standard Test Method for Direct Tensile Strength of Intact Rock Core Specimens.

[48] ASTM (2008). D3967-08: Standard Test Method for Splitting Tensile Strength of Intact Rock Core Specimens.

[49] Altindag R., Guney A. (2010). Predicting the relationships between brittleness and mechanical properties (UCS, TS and SH) of rocks. Sci. Res. Essays.

[50] Kabilan N., Muttharam M. (2016). Correlation between Unconfined Compressive Strength and Indirect Tensile Strength for Jointed Rocks. Int. J. Res. Eng. Technol..

[51] Friedheim J., Guo Q., Young S., Gomez S. Testing and Evaluation Techniques for Drilling Fluids-Shale Interaction and Shale Stability. Presented at the 45th US Rock Mechanics/Geomechanics Symposium.

[52] Byrne M.T., Spark I.S.C., Patey I.T.M., Twynam A.J. A laboratory Drilling Mud Overbalance Formation Damage Study Utilising Cryogenic SEM Techniques. Presented at the International Symposium on Formation Damage Control.

[53] Green J., Cameron R., Patey I., Nagassar V., Quine M. Use of Micro-CT Scanning Visualisations to Improve Interpretation of Formation Damage Laboratory Tests Including a Case Study from the South Morecambe Field. Presented at the European Formation Damage Conference & Exhibition.

[54] Bageri B.S., Al-Mutairi S.H., Mahmoud M.A. Different techniques for characterizing the filter cake. Presented at the SPE Unconventional Gas Conference and Exhibition.

[55] Ge X., Liu J., Fan Y., Xing D., Deng S., Cai J. (2018). Laboratory investigation into the formation and dissociation process of gas hydrate by low-field NMR technique. J. Geophys. Res. Solid Earth.

[56] Bageri B.S., Adebayo A.R., Barri A., Al Jaberi J., Patil S., Hussaini S.R., Babu R.S. (2019). Evaluation of secondary formation damage caused by the interaction of chelated barite with formation rocks during filter cake removal. J. Pet. Sci. Eng..

[57] Adebayo A.R., Bageri B.S. (2019). A simple NMR methodology for evaluating filter cake properties and drilling fluid-induced formation damage. J. Pet. Explor. Prod. Technol..

[58] Bageri B.S., Adebayo A.R., Al Jaberi J., Patil S. (2020). Effect of perlite particles on the filtration properties of high-density barite weighted water-based drilling fluid. Powder Technol..

[59] API RP 13B-2 (2014). Recommended Practice for Field Testing Oil-Based Drilling Fluids.

[60] Ombaka O. (2016). Characterization and Classification of Clay Minerals for Potential Applications in Rugi Ward, Kenya. Afr. J. Environ. Sci. Technol..

